# CMM-Net: Contextual multi-scale multi-level network for efficient biomedical image segmentation

**DOI:** 10.1038/s41598-021-89686-3

**Published:** 2021-05-13

**Authors:** Mohammed A. Al-masni, Dong-Hyun Kim

**Affiliations:** Department of Electrical and Electronic Engineering, College of Engineering, Yonsei University, Seoul, Republic of Korea

**Keywords:** Biomedical engineering, Diagnosis, Diagnostic markers, Cancer imaging, Cancer screening

## Abstract

Medical image segmentation of tissue abnormalities, key organs, or blood vascular system is of great significance for any computerized diagnostic system. However, automatic segmentation in medical image analysis is a challenging task since it requires sophisticated knowledge of the target organ anatomy. This paper develops an end-to-end deep learning segmentation method called Contextual Multi-Scale Multi-Level Network (CMM-Net). The main idea is to fuse the global contextual features of multiple spatial scales at every contracting convolutional network level in the U-Net. Also, we re-exploit the dilated convolution module that enables an expansion of the receptive field with different rates depending on the size of feature maps throughout the networks. In addition, an augmented testing scheme referred to as Inversion Recovery (IR) which uses logical “OR” and “AND” operators is developed. The proposed segmentation network is evaluated on three medical imaging datasets, namely ISIC 2017 for skin lesions segmentation from dermoscopy images, DRIVE for retinal blood vessels segmentation from fundus images, and BraTS 2018 for brain gliomas segmentation from MR scans. The experimental results showed superior state-of-the-art performance with overall dice similarity coefficients of 85.78%, 80.27%, and 88.96% on the segmentation of skin lesions, retinal blood vessels, and brain tumors, respectively. The proposed CMM-Net is inherently general and could be efficiently applied as a robust tool for various medical image segmentations.

## Introduction

Medical imaging is an approach that generates interior visual representations of the hidden internal structures inside the human body. This process could be applied noninvasively such as Magnetic Resonance Imaging (MRI), Computed Tomography (CT), X-ray, Ultrasound (US), endoscope, ophthalmoscopy, and dermoscopy modalities. Such imaging modalities play a crucial role in clinical analysis, diagnosis, and treatment planning. Computer-Aided Diagnosis (CAD) system is an indispensable tool that aims to provide assistance to clinicians through interpretations of the abnormalities existing in the medical images such as brain tumors in MR images^1^, liver nodules and pulmonary lung nodules in CT images^2,3^, breast masses in mammograms^4^, and skin lesions in dermoscopy images^5^. Most of the traditional CAD systems are performed through four consecutive stages: data preprocessing, Region of Interest (ROI) detection or segmentation, features extraction and selection, and classification. The detection stage (CADe) usually aims to localize the suspicious lesions in the input images, while the segmentation stage delineates the specific lesion boundaries. However, the diagnosis stage (CADx) utilizes the extracted features from the detected or segmented suspicious regions to differentiate between different diseases.

**Figure 1:**
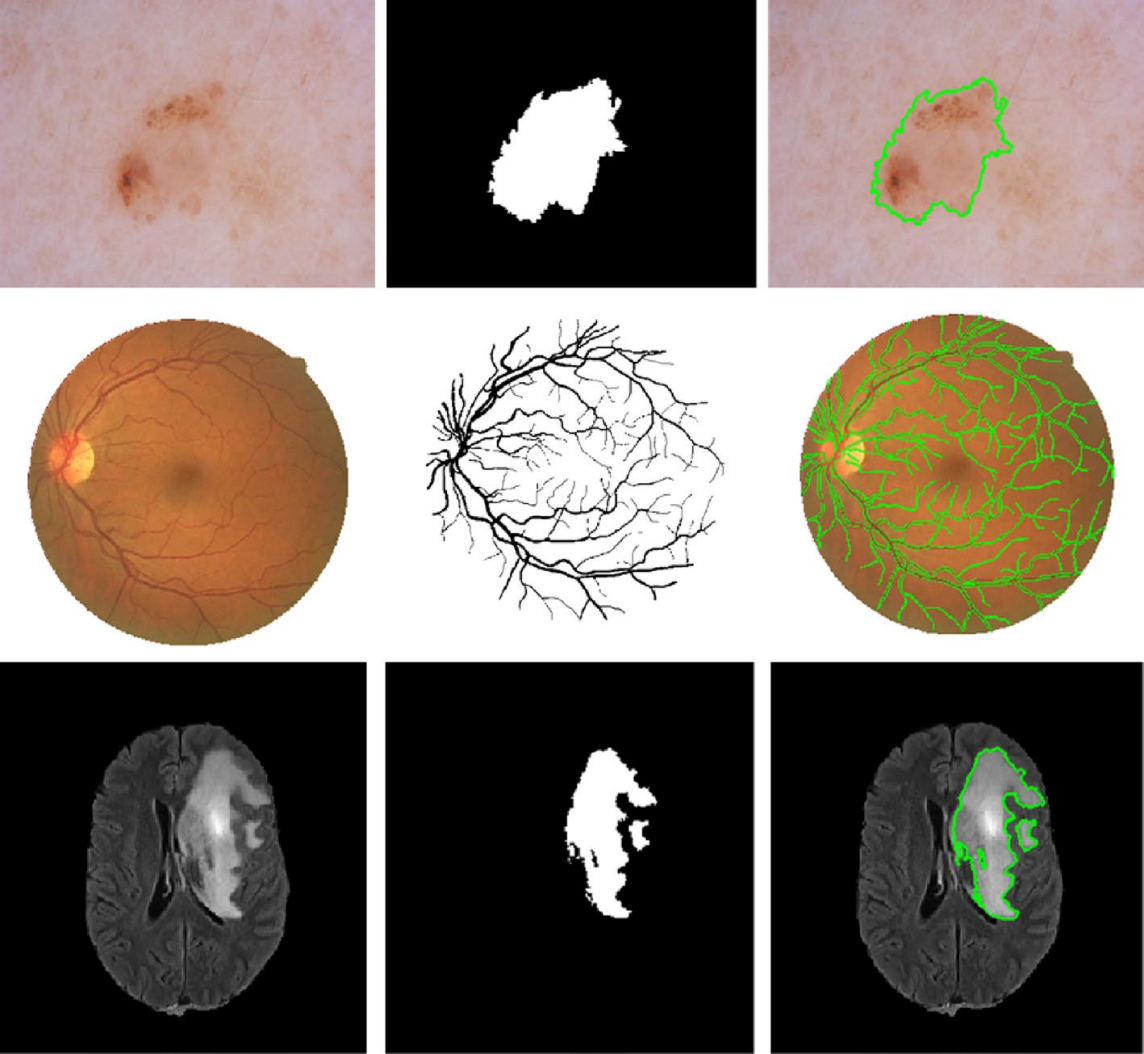
Exemplary pairs of the original medical images and their ground-truth segmentation masks.

Medical image segmentation is a fundamental preliminary step for any CAD system in medical image analysis applications. For example, the segmentation of brain tumors in MR images could considerably enable providing accurate quantitative analysis and diagnosis of ischemic stroke and Alzheimer’s diseases^6^. In addition, segmentation of blood vessels from retinal images, also known as fundus images, is of significance for automatic screening of diabetic retinopathy^7^. Similarly, skin lesion boundary segmentation using dermoscopy images is an important process to support dermatologists of recognizing melanoma from other skin cancer types in its early stages through integrating specific knowledge such as lesion’s size and contour’s shape^8^. Figure 1 illustrates some exemplary pairs of medical images and their segmentation masks such as blood vessels, brain tumors, and skin lesions (as is the case in this work). However, manual segmentation of such medical applications is a challenging task since it requires a sophisticated prior knowledge of organ anatomy. Furthermore, the annotation process itself is extremely laborious, subjective, prone to error, and time-consuming at large biomedical data. In this regard, efficient automated segmentation algorithms are highly demanded in clinical applications for more accurate analysis and diagnosis.

Even though the last decades have witnessed numerous developments for providing automated semantic segmentation methods with lower cost and less user intervention, challenges still exist in many medical imaging segmentation tasks. More specifically, the low contrast, complex geometry, irregular boundaries, high inter- or intra-class variations, as well as the presence of noise and artifact in some imaging modalities added extra obstacles to biomedical segmentation tasks. Moreover, the difficulty of medical image analysis is related to the image quality and labeling variation. One example is illustrated in Fig. 1, where small vessel segments of the retinal images are hard to be identified. Also, due to the scarcity of medical images, training as well as testing has been performed on limited image data. Different from the state-of-the-art methods, the proposed segmentation method comprises appropriate global pyramid representations at every level in the encoder network, which is also associated with multi-scale context-aware.

In this paper, we develop an end-to-end, pixel-to-pixel deep learning segmentation methodology called Contextual Multi-Scale Multi-Level Network (CMM-Net). The main idea is to fuse together the global context features of multiple spatial scales at every contracting convolutional network level in the U-Net. Also, we re-exploit the dilated convolution module that enables an expansion of the receptive field with different rates depending on the size of feature maps throughout the networks, leading to generate dense predictions of multi-scale contextual information with minimal resolution loss. Furthermore, an extension to the test-time augmentation referred to as Inversion Recovery (IR) is presented. The IR scheme is able to accumulate all the predictions of the augmented testing data using logical “OR” and “AND” operators. The proposed method achieves state-of-the-art segmentation performance on three different medical imaging modalities (i.e., skin lesions in dermoscopy images, retinal blood vessels in fundus images, and brain glioma tumor in MR images).

In the next section, we review the current approaches to semantic segmentation and previous works on medical imaging segmentation. Afterwards, the following sections describe the design of the proposed CMM-Net, the details of the utilized medical datasets, and explain the inversion recovery evaluation scheme. Finally, we present the experimental segmentation results of skin lesions, retinal blood vessels, and brain tumors.

## Related work

Recently, end-to-end deep learning segmenters based on Convolutional Neural Networks (CNNs) have been gaining attention due to their superior performance on different segmentation benchmarks. The most famous and impressive semantic segmentation methods include Fully Convolutional Networks (FCN)^9^, U-Net^10^, SegNet^11^, and DeepLab^12^. FCN was the first semantic segmentation method, which converted the conventional classification CNNs to pixel-wise segmentation by replacing the fully connected Neural Networks (NNs) layers into convolutional layers. Eventually, FCN exploited the up-convolution with the element-wise fusing from shallower layers to generate dense of predictions as the same size of the input. As an extension to FCN, U-Net, SegNet, and DeepLab were developed to improve the coarse output segmentation map by innovating encoder-decoder networks that share the hierarchy features. Lately, a lot of improvements using dilated convolution^13^, also known as atrous convolution, Spatial Pyramid Pooling (SPP)^14^, Atrous SPP (ASPP)^12^, and Pyramid Scene Parsing (PSP)^15^ have been introduced. The goal of these key ideas is to generate multi-level contextual information. For instance, dilated convolution enabled the network to control the resolution of the learned features by enlarging the receptive field of filters, equivalent to adding holes (i.e., zeros) between the elements of convolutional kernels. However, SPP eliminated the fixed input size constraint of CNN in image recognition and utilized local multi-level pooling to maintain the spatial information. In the PSP module, it was possible to incorporate hierarchical global context features from various pyramid scales to produce semantic parsing dense maps. It is noteworthy that all these components were only applied to the top layer of the network since the large pooling scales comprise more global information, while the small scales preserve the fine details.

The exploration of multi-scale feature fusion has been further studied for improvement of several semantic segmentation tasks. DeepLabv3^16^ employed the atrous convolution of various rates along with ASPP in cascade or in parallel to extract rich multi-scale contextual information. An enhanced DeepLabv3 + ^17^ was extended by adding a decoder module to refine the detailed object boundaries. A depth-wise separable convolution was also added to both the ASPP and decoder modules, producing a faster and more robust network. In addition, various extensions have been made on the encoder-decoder U-Net. Attention U-Net^18^ aggregated Attention Gates (AGs) at each decoder level. The AGs added the feature maps from the relevant encoder level with features from the former decoder level resulting in suppressing the feature responses to irrelevant regions. GridNet^19^ interconnected multiple streams or paths at different resolutions in a grid pattern. These connections were performed horizontally and vertically throughout the encoder-decoder network, leading to share low and high resolutions and hence capturing more context information for full scene labeling. A Multi-Scale Densely Connected U-Net (MDU-Net)^20^ employed the well-known DenseNets^21^ concept by combining three different multi-scale dense connections at the encoder, decoder, and cross-connections. This architecture could fuse various scale feature maps from different resolution layers. Similar to GridNet, a Full-Resolution Residual Network (FRRN)^22^ was proceeded using two processing streams. The first stream carried the full-resolution information, while the other pooling stream extracted multi-scale contexts.

More recently, large kernel SPP^23^ was proposed to address sufficient receptive fields while maintaining the same computational efficiency. This module was located at the transition layer between the encoder and decoder, where it consisted of global context network and depth-wise separable convolution. Context Encoder Network (CE-Net)^24^ was developed using three modules: encoder, context extractor, and decoder. The context extractor module contained two main blocks: dense atrous convolution block that captured deeper and wider context features by fusing cascaded paths and residual multi-kernel pooling block that encoded global context information at multi-size receptive fields. A Hyper-Densely connected CNN (HyperDense-Net)^25^ was proposed for multi-modal image segmentation. Inspired from DenseNet, HyperDense-Net densely connected not only the layers within the same path, but also these connections occurred across various paths. UNet +  + ^26,27^ extended the U-Net by redesigning the skip connections between encoder and decoder and fusing the features of different semantic scales. UNet +  + is an ensemble architecture that integrates multiple U-Nets of different depths into a single unified network. This could allow the network to aggregate the original, intermediate, and final features at decoder. As an extension to UNet +  + , UNet 3 + ^28^ replaced the nested convolution blocks at the encoder-decoder path by two different connections: densely inter-connection between encoder and decoder and densely intra-connection among decoder levels. These densely full-scale skip connections could fuse the low-level details with high-level features and maintain the full use of multi-scale features. In opposite to most existing state-of-the-art segmenters that first encoded the input into low-resolution features and then decoded and recovered the high-resolution features, High-Resolution Network (HRNet)^29^ preserved the high-resolution features throughout the whole network. This was achieved by connecting parallel multi-resolution (i.e., high-to-low resolution) convolution streams and repeating this stage with multi-resolution fusions across the parallel streams, leading to obtain richer and more precise spatial representations.

In terms of applications, deep learning networks have been employed for several medical imaging tasks including image classification^30^, object detection^31–34^, semantic segmentation^35,36^, artifact denoising^37^, and image reconstruction^38^. Also, many studies have been conducted for skin lesion boundary segmentation^8,35,39–41^, retinal blood vessel segmentation^42–44^, and brain tumor segmentation^45–48^. Further, some works have been conducted for multiple medical image segmentation^24,26,27,49,50^.

In addition to training data augmentation, some deep learning researches have presented the effectiveness of Test-Time Augmentation (TTA), in which the following steps were used: test data augmentation, prediction, and merging or averaging of the results. Krizhevsky et al.^51^ and Simonyan et al.^52^ averaged the predictions of multiple cropped patches around the object and horizontally flipped images to obtain the final score of an image classification model. These multiple predictions of a given test image help to achieve more robust inference. More recently, this procedure has been applied to medical image segmentation tasks to improve segmentation accuracy. The final segmented label was computed as an average or as a pixel-wise majority voting of the predicted pixels^53–57^. The disadvantage of TTA is its computational cost since the inference is performed many times depending on the number of augmentations. However, TTA is promising for medical image applications.

### Our contributions

The main contributions of this paper are fourfold.

- We propose an end-to-end deep learning network for medical image segmentation named Contextual Multi-Scale Multi-Level Network (CMM-Net). The main idea of our CMM-Net is to generate global multi-level contextual information at every encoder convolutional level, which allows the network to learn various spatial scales of the target with minimal resolution loss. This is achieved by frequently promoting pyramid pooling and dilated convolution modules with different spatial rates throughout the networks.
- The proposed work achieves state-of-the-art performance on three different medical imaging benchmarks, including segmentation of skin lesion from dermoscopy images, retinal blood vessels from fundus images, and brain malignant glioma from MR images. We compare our results to recent state-of-the-art architectures such as U-Net, PSP-Net, DeepLabv3 + , CE-Net, and UNet +  + .
- We adopted a modified segmentation evaluation scheme called Inversion Recovery (IR), which can generate more accurate segmentation maps by utilizing augmented testing data using a combination of logic operations.
- We make the source code publicly available for researchers for validation and further improvement here: https://github.com/Yonsei-MILab/Biomedical-Image-Segmentation-via-CMM-Net*.*

## Materials and methods

### Dilated convolution

Dilated convolution or atrous convolution^13^ is inspired by wavelet decomposition^58^, which is able to assemble multi-scale contextual features instead of using successive pooling layers. It controls the resolution of such information by exponentially enlarging the receptive field of filters. Consider $f[x]$ be a discrete input and $w[x]$ be a discrete filter or kernel. The standard spatial convolution can be computed as:

1 $$ f\left[ x \right]*w\left[ x \right] = \mathop \sum \limits_{k = - \infty }^{ + \infty } f\left[ k \right] \cdot w\left[ {x - k} \right] , $$

where $^{\prime}*^{\prime}$ and $^{\prime} \cdot ^{\prime}$ indicate the convolution and ordinary multiplication operations, respectively. Then, the dilated convolution with dilation rate $r$ is defined as:

2 $$ f\left[ x \right]*_{r} w\left[ x \right] = \mathop \sum \limits_{k = - \infty }^{ + \infty } f\left[ k \right] \cdot w\left[ {r\left( {x - k} \right)} \right] . $$

**Figure 2:**
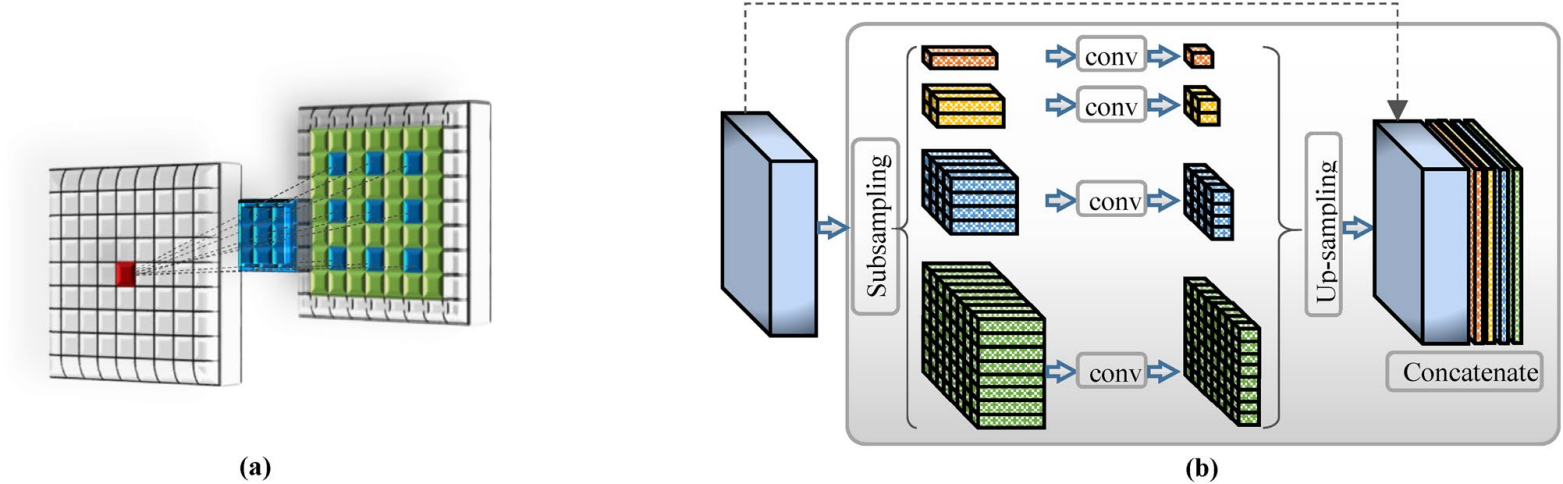
(**a**) An example of dilated convolution at rate of two, which generates a receptive field of 7 $\times$ 7 pixels. (**b**) Pyramid Pooling Module (PPM) configuration.

It is of note that the resulting receptive field expands exponentially when the dilation rate gets increased. This relation can be formulated as $\left( {2^{r + 1} - 1} \right) \times \left( {2^{r + 1} - 1} \right)$, in which it becomes identical to standard convolution when $r = 1$. Thus, the dilated convolution could control the resolution of the contextual information where the number of learning parameters increases linearly. Figure 2a depicts the concept of the dilated convolution with its related receptive field.

### Pyramid pooling module (PPM)

The key role of Pyramid Pooling Module (PPM) is to generate ensemble high-level feature maps, which represent global context information of multi-scales. In contrast to the SPP that passes the flattened and concatenated multi-level features into FC-NN in the classification tasks, PPM could reduce the loss of information between various sub-levels and extract effective hierarchical global representations. Figure 2b illustrates the configuration of the PPM. The PPM starts with subsampling the convolved features into four parallel pyramid levels with different scales. Larger pooling factor produces coarser features (i.e., similar to global average pooling), while the finer representations are extracted with smaller pooling factors. Then, the bottleneck layer that uses 1 $\times$ 1 convolution is applied directly after each pooled features to improve the computation capability by reducing the context dimension to $1/N$, where $N$ indicates the pyramid’s level size. For instance, if the level size of the pooling pyramid $N=4$ (as the case of this work), then the feature maps of each level will be reduced by the factor of $1/4$. To get back to the original feature maps right before the pyramid pooling, up-sampling via bilinear interpolation is applied to each pyramid level. Eventually, concatenation of all the up-sampled feature maps with the original feature map is conducted to fuse global context features.

The above two key modules have been exploited to design our proposed network as explained in the following section.

### Proposed CMM-Net architecture

**Figure 3:**
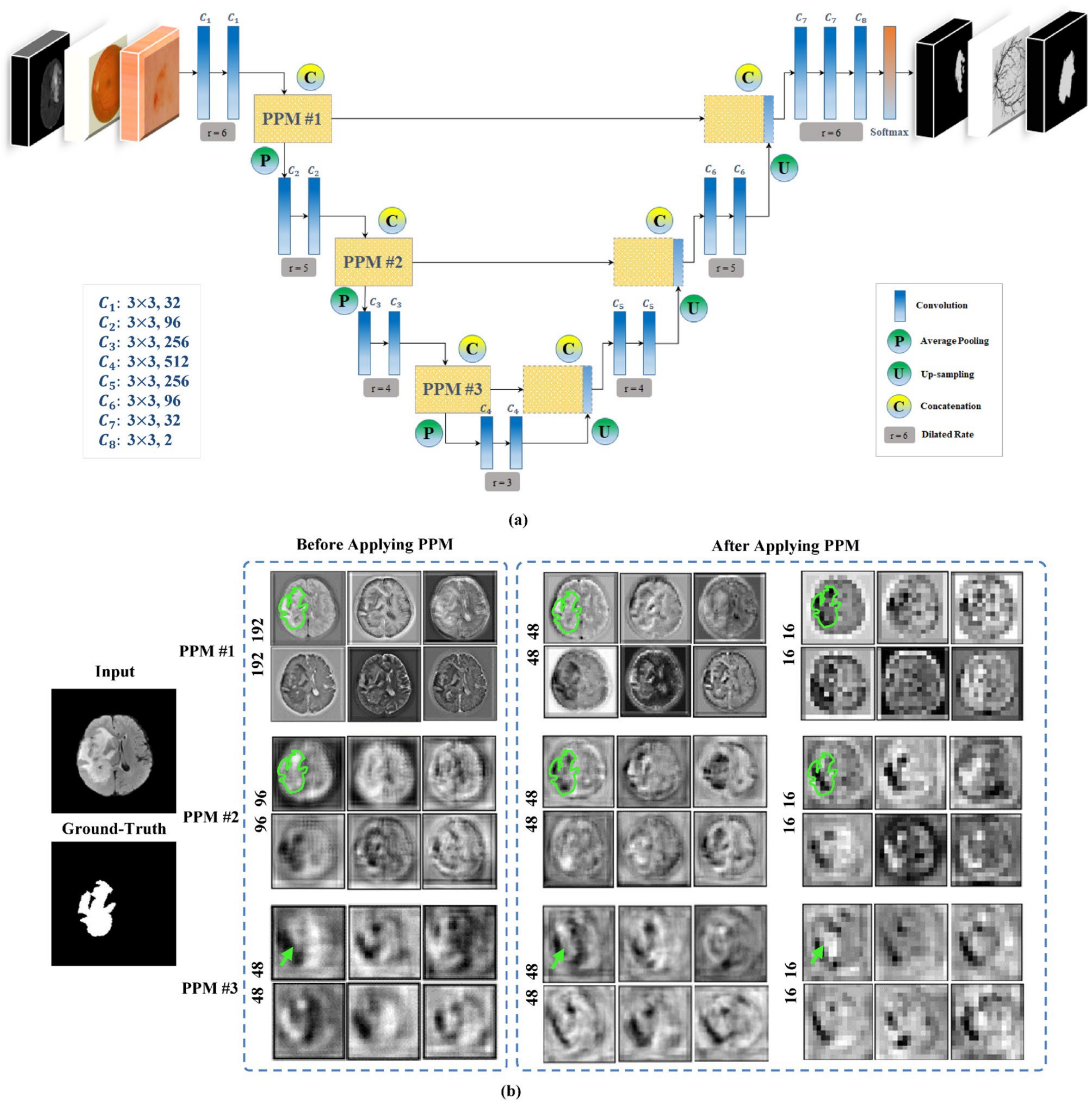
(**a**) The architecture of the proposed CMM-Net segmentation method. Details of the filter sizes and the number of feature maps are illustrated in the bottom left corner of (**a**). (**b**) Visualization of example feature maps before and after applying the PPM at different encoder levels. The larger two scales of each PPM with sizes of 48 $\times$ 48 and 16 $\times$ 16 are presented since they contain intelligible patterns.

Inspired by deep learning U-Net^10^, we propose an end-to-end segmentation network called CMM-Net. Similar to the original U-Net, the proposed network consists of contracting and expanding paths, also known as encoder and decoder networks. The contracting path contains sequential convolutional and subsampling layers, which are responsible to capture hierarchical features. Symmetrically, the expanding path involves up-convolution and up-sampling layers, in which all the features in the encoder are passed and concatenated with the feature maps in the decoder. This process increases the resolution of the output’s dense map and hence allows for more accurate localization. In this work, the exploited U-Net consists of four main levels, including eight convolutions and three pooling layers in the contracting path and seven up-convolutions, three up-sampling, and a single softmax layer in the expanding path. Details of the feature maps and convolution filter sizes are presented in Fig. 3.

Unlike previous semantic segmentation methods such as PSPNet^15^, Deeplabv3 + ^17^, large kernel SPP^23^, and CE-Net^24^ that applied pyramid pooling at only the top of encoder network (i.e., only PPM #3 block in Fig. 3a), the proposed work suggests to make full utilization of the pyramid pooling module. This is accomplished by repeatedly attaching the module at each level in the encoder network (i.e., PPM #1, 2, and 3) as shown in Fig. 3a. Thus, the redesigned frequent pyramid pooling network enables to extract and learn multi-spatial global information and allows to share them between encoder and decoder networks. Also, different from GridNet^19^, UNet +  + ^26,27^, and UNet3 + ^28^ that re-designed the plain skip connections in the original U-Net by strengthening the encoder-to-decoder gap using wired, nested convolution blocks, or densely connections, which cause increase in the number of trainable parameters, the proposed network aggregates multi-scale global representations at various skip connection levels while maintaining lower computational cost. The novelty of the proposed work is based on the way that the utilized components (i.e., dilated convolution and PPM) are re-designed to make full use of global multi-scale contextual information through each network layer, which leads to capturing various spatial scales while minimizing the input resolution loss. This fusion scheme of the multi semantic scales enables to share coarse-to-fine details with localization information at every level between the encoder and decoder. More specifically, applying the PPM at the early encoder level, as the case of PPM #1 and 2, results in learning global fine-grained and structural details as well as preserving the input resolution, while the PPM at the top of the encoder (i.e., PPM #3) extracts coarser representations. Figure 3b visualizes example feature maps before and after applying the PPMs. It is shown how efficient PPM #1 is in extracting global features while maintaining input resolution with location-awareness compared to the feature maps after PPM #3. The green contours in Fig. 3b represent the precise features of the target in the image, which are intelligible within the feature maps of PPM #1 and 2, while the green arrows indicate the coarser information at PPM #3. This strategy enables the proposed network to achieve efficient multi-scale multi-level segmentation.

To achieve superior segmentation performance, we have adopted three PPM units, involving four different scales varied in the range between 1 $\times$ 1 to 64 $\times$ 64 pixels, while the size of feature maps in each level of PPM is set to be 64 in the case of skin lesion and brain tumor datasets and 128 in the case of retinal blood vessels data. In this work, we deal with different medical image modalities that have various input sizes. Due to that, the larger pooling scale of 64 $\times$ 64 is applied with the skin lesion dermoscopy data, however, 48 $\times$ 48 and 32 $\times$ 32 are set for brain tumor MR and retinal fundus images, respectively. It is of note that all these parameters of the PPM can be modified.

Furthermore, we have employed the dilated convolution strategy in all designed convolutional layers. To address the input size variation throughout the network (i.e., due to the existence of pooling layers), we assign different dilated rates, which directly related to input sizes. More specifically, the dilated rate $r$ is set to be 6, 5, 4, and 3 in the encoder levels, while it is 4, 5, and 6 in the decoder levels as noted in Fig. 3. Hence, larger receptive fields are obtained to derive high-resolution feature maps.

All the convolutional layers in the proposed network have been appended by batch normalization (BN) process and activation function (ReLU). The extracted prominent representations at the last convolutional layer are fed into a softmax function. In our task, the softmax layer works as a binary classifier since each pixel is categorized into tumor or tissue as the case of skin and brain datasets and into vessel or background as the case of retinal images.

### Network training

In this work, we have conducted network training using the double cross-validation strategy^59^. The key role of the double cross-validation is to determine the tunable parameters and avoid any bias procedure during building the model. This process is accomplished by dividing the data into three subsets, namely the training, validation, and testing sets. The network optimization (i.e., selecting proper hyper-parameters) is performed using the validation set, while the model evaluation is proceeded using the testing set. During network training, forward and backward propagation cycles are occurred, leading to compute the prediction maps and estimated segmented errors. The estimated error between the segmented maps ($SM$) and the ground-truth annotations ($GT$) is computed using dice loss function $\left( L \right)$ as follows,

3 $$ L = 1 - \frac{{2 \times \left( {SM \cap GT} \right)}}{SM \cup GT}{ }. $$

The proposed CMM-Net is trained using Adam optimization method with a batch size of 20 for both skin lesion and retinal fundus datasets and 5 for the brain tumor dataset. This variation is due to the hardware requirements of the GPU memory limit, where the brain tumor dataset contains very large augmented training images. Moreover, we initially set the learning rate to 0.0001 and it is reduced by a factor of 10 throughout 100 epochs for both skin cancer and retinal fundus tasks and 30 epochs for brain glioma MR data.

The implementation of this work is conducted using a PC equipped with GPU of NVIDIA GeForce GTX 1080 Ti. This work is implemented using Python programming language, Keras library, and Tensorflow backend.

### Inversion recovery (IR) evaluation scheme

**Figure 4:**
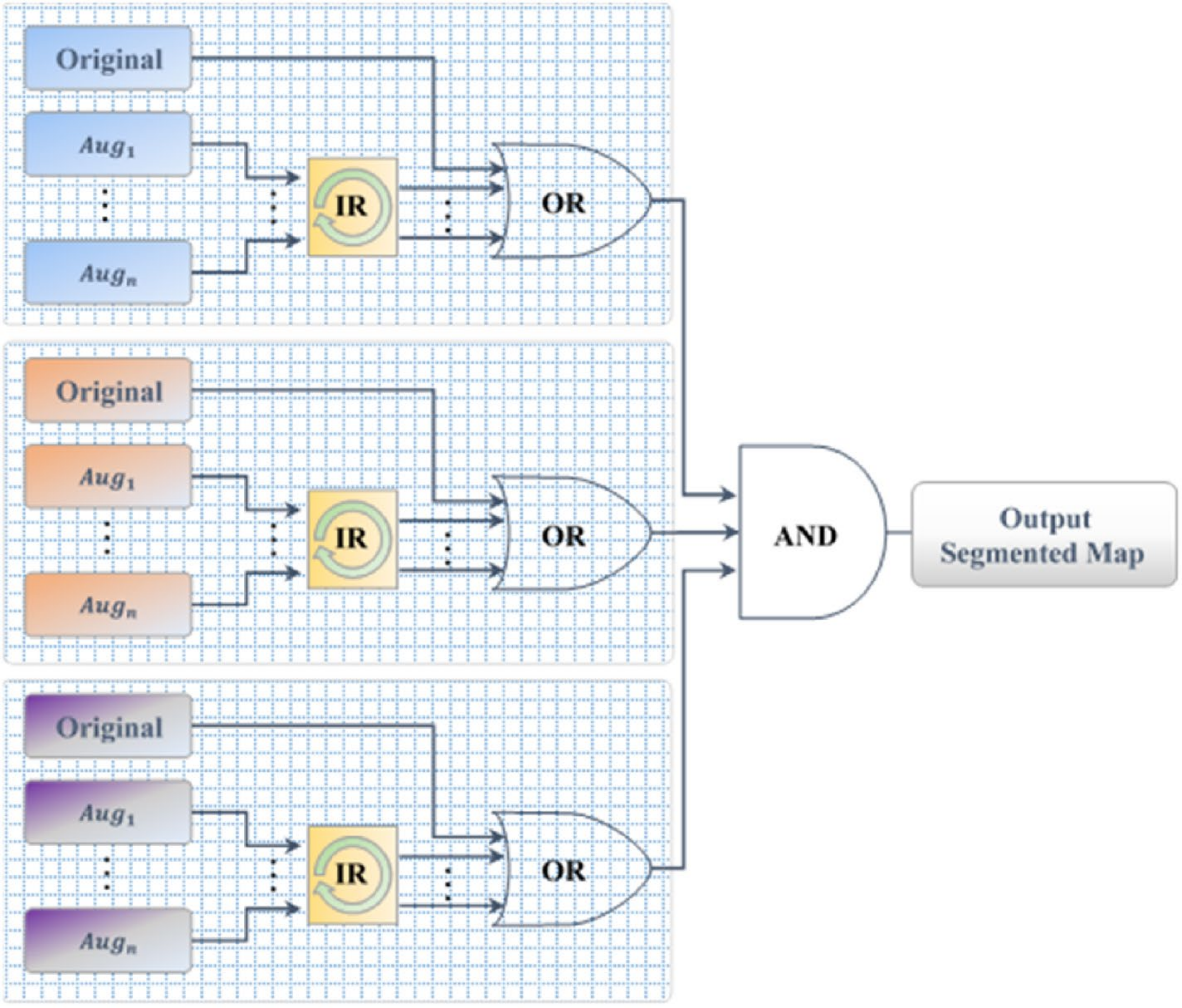
Inversion recovery evaluation scheme.

In this study, we present a version of TTA for image segmentation tasks called Inversion Recovery (IR), which is performed as a post-processing step applied to augmented test data. The main idea is to accumulate different segmented maps of various orientations for the same original testing image. This process requires to first augment each testing data multiple times using different rotations and flipping. After these augmented data have been tested via the proposed CMM-Net, we retrieve their prediction maps (i.e., segmented masks) into the original orientation by applying inverse augmented processes. Then, we fuse all the retrieved segmented maps along with the original map utilizing the logical “OR” and “AND” operators. In other words, for a given test image, multiple predictions or segmentation maps can be generated based on applying different augmentations to each test image. The final segmented result is computed as an ensemble of their prediction maps. This procedure allows to get more confident results as well as improving the overall segmentation performance. We illustrate the IR evaluation scheme in Fig. 4. Different from TTA that creates multiple samples of each test data, then returning a single ensemble prediction by averaging the results of all augmented copies per sample, the proposed IR performs this process using logical “OR” and “AND” operators. It is assumed that this IR scheme matches well with the CMM-Net due to its multi-scale learning ability. The motivation to use this augmented IR scheme is that the multi-scale learning filters of the CMM-Net can be expressed efficiently when tested with different views.

Formally, consider $I_{o}$ is the segmented binary map for any original test image. This map is updated using an “OR” operator with other segmented maps $I_{k}$ of the recovered augmented data for the same original image. Then, the resulting new segmented map can be identified as follows,

4 $$ I_{o} = \left( {I_{o} | IR\left( {I_{k} } \right)} \right) , \quad k = 1, \ldots ,n, $$

where ‘|’ refers to the “OR” operator and $n$ indicates the number of augmented maps. Further, the “AND” operator is utilized to accumulate results when multi-color space is employed as the case of skin lesion segmentation. This proposed evaluation scheme could improve the segmentation performances of various medical imaging applications.

## Experiments

### Databases and their preparation

In this work, three public medical imaging benchmarks were utilized to evaluate the proposed CMM-Net segmentation method. Example images with their ground-truth segmentation masks are demonstrated in Fig. 1.

#### Skin Lesions

The most widely utilized and available database for skin lesion segmentation is known as the International Skin Imaging Collaboration (ISIC) Challenge 2017. The ISIC 2017 dataset involves a total of 2,750 RGB dermoscopy images, which are divided into 2,000 samples for training, 150 samples for validation, and 600 samples for testing. All the provided dermoscopy images are paired with the binary segmentation masks, which were delineated by expert dermatologists.

Due to the large variation in image sizes ranged from 540 $\times$ 722 to 4499 $\times$ 6748 pixels, we rescaled all the data to 192 $\times$ 256 pixels, as a prerequisite step, using bilinear interpolation as successfully applied in^8^.

#### Retinal blood vessels

The experiments of how efficient our proposed CMM-Net on segmenting the retinal blood vessels are proceeded utilizing the Digital Retinal Images for Vessel Extraction (DRIVE) dataset^60^. The DRIVE dataset consists of 20 retinal images for training and 20 images for testing obtained from the screening program for diabetic retinopathy in The Netherlands. This dataset is provided along with manually segmented annotations of the retinal vasculature by expert ophthalmologists and binary masks of the field of view (FOV). The DRIVE dataset contains 8-bit RGB images with a fixed size of 584 $\times$ 565 pixels.

To improve the visibility of the retinal images and in particular the small vessels, we applied the Contrast Limited Adaptive Histogram Equalization (CLAHE) method^61^. Further, due to the limited size of this dataset, we considered a patching approach to enlarge the number of training samples. Firstly, we cropped the input data that exists in the FOV. Then, we extracted overlapped small patches from all training retinal images with a size of 128 $\times$ 128 pixels and a stride of 64, resulting in 1,280 patches. However, for the testing data, 500 non-overlapped patches were extracted.

#### Brain tumors

Multimodal Brain Tumor Segmentation Challenge (BraTS) is the largest and publicly available dataset, which focuses on segmenting the brain tumors, namely gliomas (low- and high-grade), from multi MRI modalities^62–64^. In this paper, we utilized the BraTS 2018 dataset, which contains 285 training cases and separate sets of 66 and 191 cases for validation and testing, respectively. It is of note that, the ground-truth annotations of the training cases are only accessible. However, it is possible to evaluate the proposed segmentation network using the validation cases throughout the online submission system. Regarding the testing set, it was only utilized to rank participants at the challenge time. Thus, we have randomly split the original training cases into 80% for network training and the rest 20% for a network assessment. In this paper, we call this 20% set as a local test data. Each case in this dataset includes four 3D MRI modalities, named as T1, contrast T1-weighted (T1Gd), T2-weighted, and T2 Fluid Attenuated Inversion Recovery (FLAIR), with a size of 240 $\times$ 240 $\times$ 155 voxels. In spite of that the tumors in the BraTS 2018 dataset are categorized into the Whole Tumor (WT), Enhancing Tumor (ET), and Core Tumor (TC), in this work we focused on only segmenting the WT as further evaluation of the efficiency of the proposed CMM-Net.

To reduce the network computations, we cropped the volumes to only contain brain data to 192 $\times$ 192 $\times$ 155 voxels. In addition, we generated three-channel input images using T2, FLAIR, and T1Gd. This could increase the visibility of the brain tumors, leading the network to extract more robust features. For the training data generation, we collected all input 2D slices that contain ground-truth annotations (i.e., gliomas), resulting in 15,290 labeled images. However, at evaluation time, all the slices in the testing and validation sets were passed to the network.

The original data distributions of the utilized medical imaging databases are summarized in Table 1. As aforementioned, the numbers of the training and local testing cases of the BraTS 2018 that shown in this table are summed up to 285 cases, which represents the original training data.

**Table 1:** Distribution of the three medical imaging databases. ‘*’ Indicates to the local training and testing sets of the BraTS 2018 dataset.

Database	Type	Training	Validation	Testing
ISIC 2017	Images	2000	150	600
DRIVE	Images	20	–	20
	Patches	1280	–	500
BraTS 2018	Volume	228*	66	57*
	Images	15,290	10,230	8835

All datasets are normalized between zero and unity. Moreover, to enlarge the training sets, which enables appropriate learning of the network and reduces the overfitting problem, we augmented all the databases eight times (i.e., including the original ones) using different rotation and flipping processes. An exception, we further utilized different color space features such as LAB (L for lightness, A for red-green value, and B for blue-yellow value) and SV (S for saturation and V for Value) besides the RGB in the case of skin lesion database since it helps on illustrating more details.

### Evaluation metrics

The proposed CMM-Net segmentation method is quantitatively evaluated using several performance evaluation measures, including sensitivity (SEN), specificity (SPE), accuracy (ACC), Dice similarity coefficient (DSC), Jaccard (JAC) index, and Matthew correlation coefficient (MCC). Moreover, we utilized the receiver operator characteristic (ROC) curve along with its area under the curve (AUC) as well as precision-recall (PR) curve. For the definitions and formulas of all these indices, refer to this article^35^.

### Experiments setup

This section experiments the effect of key components of the proposed CMM-Net method on the skin lesion boundaries segmentation. It includes the segmentation of the original U-Net as a baseline, repeated PPMs, dilated convolution, augmentation of training data, and the IR evaluation process. In this experiment, we utilized the Jaccard index to assess the improvement of each added component since it was utilized by the ISIC 2017 challenge to rank the participants. Table 2 presents the segmentation performances when adding different components to the original U-Net. It is noteworthy that all these investigations were conducted using the same conditions and hyper-parameters. Starting with the baseline U-net, it obtained a Jaccard index of only 67.36% for overall skin lesions segmentation. The experiments of gradually applying the PPM #1, 2, and 3 demonstrate the effectiveness of generating multi-level contextual information. Applying the PPM #1 improves the segmentation performance by 1.16% in term of Jaccard index, while training the network using both PPM #1 and #2 improves the performance with an incremental rate of 2.49%. A significant increment of 5.46% was achieved in term of the Jaccard index in the case of adding the PPMs to all the contractive levels of the original U-Net. Again, a marginal increment from 72.82% to 73.94% was obtained when training the proposed network with the dilated convolutions. All of these investigations were applied with the original training images. Regarding the effect of training the proposed CMM-Net using the augmented training data, a marginal increment of 0.54% in term of the Jaccard index was achieved for overall skin lesion boundaries segmentation. It is of note that the aforementioned experiments were evaluated using the original test images (i.e., without augmented the test set). Finally, we showed the effect of using the IR evaluation scheme, which requires to first augment the testing images. This process enables the proposed network to segment the skin lesions with a higher incremental rate of 3.17% compared to the previous investigation. Generally, our proposed CMM-Net method achieved superior performance on segmenting the skin lesions with an overall Jaccard index of 77.65% compared to baseline original U-Net that obtained 67.36%. This implies that an improvement of a total of 10.29% was gained via our proposed work. It is observed that the sensitivity in some experiments is degraded. This degradation is probably related to the relatively increasing of the amount of false negatives (i.e., lesion pixels that were segmented falsely as non-lesions).

**Table 2:** Evaluations of different network’s component setup.

Network setup	SEN	SPE	ACC	MCC	DSC	JAC
Original U-Net	82.69	94.34	90.58	73.43	77.40	67.36
+ PPM #1	78.20	97.02	90.88	74.43	77.44	68.52
+ PPM #1 and #2	76.06	**97.89**	91.33	75.52	78.38	69.85
+ All repeated PPMs	79.86	97.56	91.98	78.65	81.59	72.82
+ Dilated Convolution	81.60	97.07	92.26	79.64	82.95	73.94
+ Augmented Training Data	82.40	97.44	92.64	80.28	83.13	74.49
+ IR Evaluation Scheme	**87.69**	96.23	**93.93**	**82.61**	**85.78**	**77.65**

As presented, we observed that all the proposed components in the network contributed to improve the segmentation performance. In the next section, we present the experimental results of our proposed CMM-Net on three different medical imaging tasks. To provide a further comparison, we implemented state-of-the-art segmentation methods such as U-Net, PSPNet based on VGG network, DeepLabv3 + , CE-Net, and UNet +  + using the same augmented training data. For all methods, we showed the segmentation results with and without applying the IR process.

## Results and discussion

### Segmentation results on various applications

#### Skin lesion segmentation performance

This section presents the performance of the proposed CMM-Net method on segmenting the skin lesions using 600 test dermoscopy images of the ISIC 2017 dataset. Quantitatively, we report the experimental results of our proposed work against the latest state-of-the-art techniques^65–71^ in Table 3. The proposed CMM-Net method achieved superior segmentation performance compared to others. It outperformed the first ranked method (i.e., deep CDNN^65^) in the ISIC 2017 challenge by 5.19%, 0.53%, 0.88, and 1.15% in terms of overall segmentation sensitivity, accuracy, DSC, and Jaccard indices, respectively. It is of note that all the listed methods in Table 3 utilized the same test data. The experimental results showed that our CMM-Net achieved superior DSC and Jaccard scores of 85.78% and 77.65, respectively, while maintaining a high true positive segmentation rate of 87.69%. In contrast, the proposed segmentation method obtained moderate specificity and overall accuracy indices of 96.23% and 93.93% compared to other studies, respectively. The proposed method outperformed the U-Net, PSPNet, DeepLabv3 + , CE-Net, and UNet +  + by 6.62%, 4.63%, 1.52%, 2.43%, and 1.2% in term of Jaccard index, respectively.

**Table 3:** Skin lesions segmentation performances (%) via the proposed CMM-Net compared to the recent deep learning approaches.

Method	Parameters	Implemented	IR	SEN	SPE	ACC	MCC	DSC	JAC
Deep CDNN^65^(1st place in the challenge)	5.0 M	×	×	82.50	97.50	93.40	–	84.90	76.50
LIN^66^	–	×	×	85.50	97.40	95.00	–	83.90	75.30
DAGAN^67^	–	×	×	83.30	97.50	93.10	–	85.10	76.90
DSNet^68^	10.0 M	×	×	87.50	95.50	–	–	–	77.50
CSARM–CNN^69^	–	×	×	80.22	**99.40**	**95.85**	82.32	84.62	73.35
DDN^70^	–	×	×	82.50	98.40	93.90	–	**86.60**	76.50
DRN^71^	69.8 M	×	×	–	–	94.50	–	86.20	76.80
U-Net^10^	7.3 M	✓	×	**90.81**	90.09	90.89	73.40	77.94	68.02
			✓	77.93	97.48	91.78	76.62	79.85	71.03
PSPNet^15^	15.2 M	✓	×	90.58	93.78	92.88	78.46	82.55	72.52
			✓	81.69	97.04	92.52	78.92	82.51	73.02
DeepLabv3 + ^17^	2.1 M	✓	×	83.27	97.13	93.02	80.79	83.98	75.29
			✓	89.39	95.50	93.42	81.49	84.86	76.13
CE-Net^24^	26.0 M	✓	×	86.06	96.63	92.82	80.62	83.74	74.95
			✓	82.74	97.48	92.83	80.85	83.79	75.22
UNet + + ^27^	24.2 M	✓	×	90.49	94.53	93.44	81.08	84.72	76.13
			✓	88.60	95.79	93.58	81.46	84.86	76.45
Proposed CMM-Net	10.2 M	✓	×	82.40	97.44	92.64	80.28	83.13	74.49
			✓	87.69	96.23	93.93	**82.61**	85.78	**77.65**

CDNN: Convolutional-Deconvolutional Neural Networks; LIN: Lesion Indexing Network; DAGAN: Decision Augmented Generative Adversarial Networks; DSN: Dermoscopic Skin Network; CSARM-CNN: Channel & Spatial Attention Residual Module; DDN: Dense Deconvolutional Network; and DRN: Dense-Residual Network. The results of U-Net are different from those presented in Table 2 (i.e., first row) because the network was trained using the augmented training data similar to other methods in this table.

**Figure 5:**
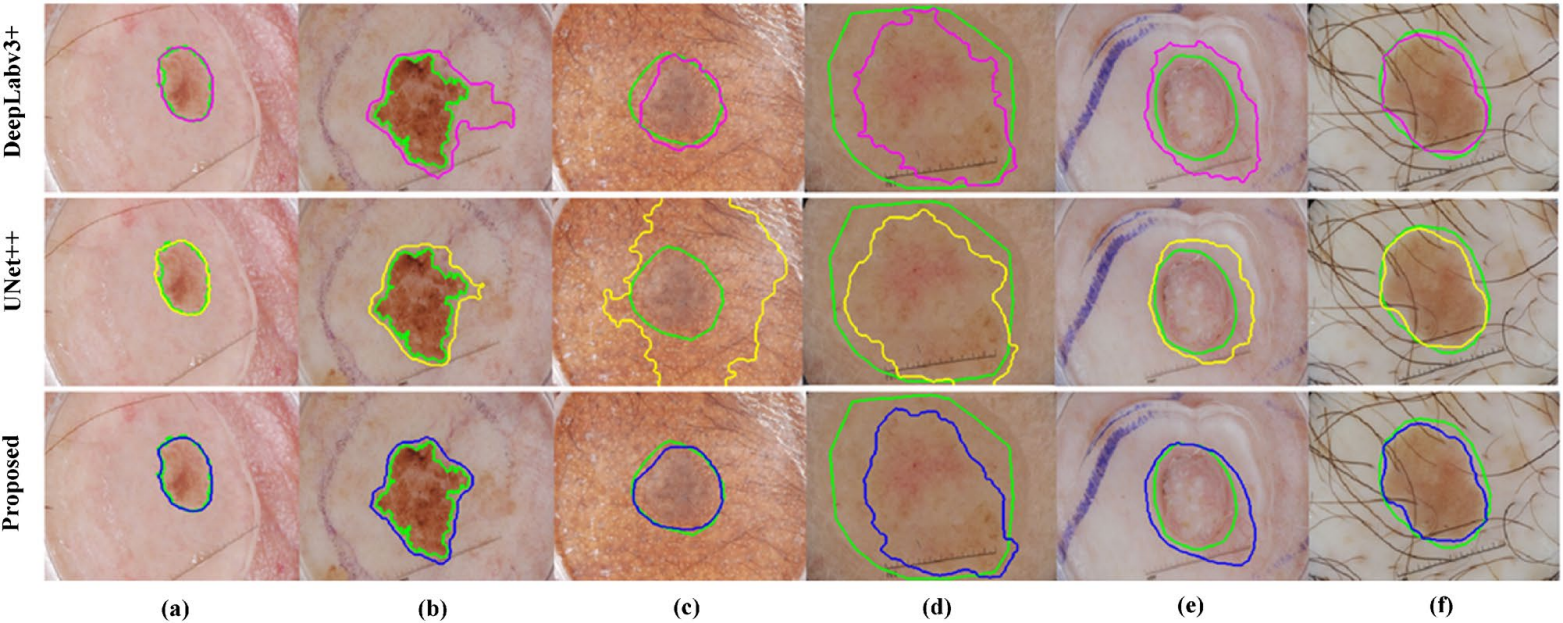
Examples of the segmented skin lesions via the proposed CMM-Net (blue contours) against the ground-truth annotations (green contours). (**a**)–(**c**) Represent precise segmentation results, (**d**) and (**e**) refer to under-fitting and over-fitting segmented samples, respectively, and (f) indicates accurate boundary segmentation result with the presence of the hair artifact. The segmentation results of DeepLabv3 + (magenta contours) and UNet +  + (yellow contours) are presented in first and second rows, respectively.

Qualitatively, some examples of the segmented skin lesion boundaries via our CMM-Net (blue contours) compared to the ground-truth annotations (green contours) are illustrated in Fig. 5. The results showed how efficient our proposed network is on segmenting the irregular skin lesions. Figure 5a–c shows accurate segmentation of the skin lesion boundaries. As demonstrated in Fig. 5d and e, the proposed CMM-Net seems to have the ability to segment the suspicious regions with high visual similarity to the lesions, causing under-fitting and overfitting of the segmented contours compared to the ground-truths. Such cases need to be reconfirmed from the specialists in the field. Moreover, our proposed method has the capability to segment some challenging cases, like lesions that exist within hair artifact as the case shown in Fig. 5f. We also show the segmentation results of the same examples via DeepLabv3 + (magenta contours) and UNet +  + (yellow contours) in the first and second rows of Fig. 5, respectively.

#### Retinal blood vessel segmentation performance

**Figure 6:**
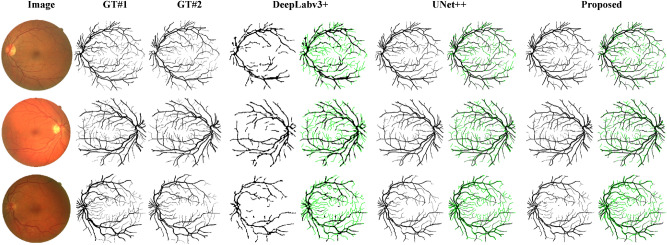
Examples of the segmented retinal blood vessels. The first column to the last column refers to original retinal images, first ground-truth (GT#1), second ground-truth (GT#2), and the segmentation results via DeepLabv3 + , UNet +  + , and proposed CMM-Net. The segmented results with the missing retinal vessels are drawn in green contours for all methods.

In this section, we further evaluated our proposed CMM-Net on extracting the retinal vasculature using the 20 fundus images of the DRIVE test dataset. Despite that the segmentation was proceeded using the 500 non-overlapped patches that were generated from the original 20 test images, the segmentation performance was evaluated based on the original image-level after restructuring the small patches into the full segmented images. The overall segmentation performance of the retinal blood vessels via our proposed CMM-Net is summarized in Table 4. It is shown that the proposed method achieved eminent segmentation performance compared to the latest deep learning approaches^42,43,49,50,72–77^ with overall MCC, DSC, and accuracy scores of 78.57%, 80.27%, and 96.64%, respectively. In contrast, MCGU-Net^50^ achieved a higher DSC for the segmentation of the retinal blood vessels at 82.24%. Since the target in this task is small and thin segments of blood vessels, we found that training the proposed network without using dilated convolution (i.e., $\mathrm{r}$=1) could achieve better extraction of blood vessels with an incremental rate of 1.88% in terms of DSC with the usage of IR. As expected, a smaller receptive field is beneficial for thinner targets. Further, the proposed method obtained better segmentation performance with DSC of 80.27% compared to DeepLabv3 + and CE-Net that achieved 62.97% and 79.60%, respectively. However, the UNet +  + achieved slightly better results compared to the proposed method with a marginal increment of 0.25% in term of DSC. This may be due to the intensive use of the nested convolution blocks in UNet +  + with increased number of trainable parameters of 24.2 M compared to 13.2 M in our case. Regarding the implemented PSPNet, it failed to segment the fragments of the retinal blood vessels. This may due to the small number of the target vessels’ pixels compared to the background tissue in the training patches. As known, the DRIVE dataset includes two different ground-truths by two experts. When comparing their segmentations (i.e., one as labeled and other as segmented), the overall sensitivity of 77.57%, DSC of 78.79, and an accuracy of 96.37% were obtained, have a look at second and third columns of Fig. 6.

**Table 4:** Retinal blood vessels segmentation performances (%) via the proposed CMM-Net compared to the recent deep learning approaches.

Method	Parameters	Implemented	IR	SEN	SPE	ACC	MCC	DSC	JAC
U-Net + joint losses^43^	–	×	×	76.53	98.18	95.42	–	–	–
FCN^42^	–	×	×	80.39	98.04	95.76	–	–	–
R2U-Net^49^	1.0 M	×	×	77.92	98.13	95.56	–	81.71	–
MCGU-Net^50^	–	×	×	80.12	97.86	95.6	–	**82.24**	–
Thick-Thin-Fusion Net^72^	–	×	×	76.31	98.20	95.38	–	**–**	–
AA-UNet^73^	28.3 M	×	×	79.41	97.98	95.58	–	82.16	–
DDNet^74^	56.0 M	×	×	81.26	97.88	95.94	–	**–**	**–**
VGN^75^	7.91 M	×	×	93.82	92.55	92.71	–	–	**–**
MTL^76^	–	×	×	**96.90**	92.70	94.70	–	**–**	**–**
SUD-GAN^77^	–	×	×	83.40	98.20	95.60	–	**–**	**–**
U-Net^10^	7.3 M	✓	×	50.50	97.96	93.34	57.22	59.61	42.46
			✓	53.98	97.90	94.11	58.78	61.27	44.17
PSPNet^15^	15.2 M	✓	×	13.92	**98.94**	91.49	24.74	22.12	12.48
			✓	16.40	98.80	91.57	27.19	25.24	14.49
DeepLabv3 + ^17^	2.1 M	✓	×	56.71	97.39	93.82	58.58	61.50	44.45
			✓	78.69	93.20	91.92	60.24	62.97	46.01
CE-Net^24^	26.0 M	✓	×	72.24	98.88	96.53	76.95	78.33	64.45
			✓	79.55	98.10	96.45	77.81	79.60	66.14
UNet + + ^27^	24.2 M	✓	×	89.27	96.90	96.21	78.92	80.42	67.29
			✓	77.26	98.64	**96.75**	**78.98**	80.52	**67.44**
Proposed CMM-Net (With Dilated Convolution)	13.2 M	✓	×	69.66	98.92	96.35	75.50	76.82	62.43
			✓	84.38	97.08	95.95	76.51	78.39	64.49
Proposed CMM-Net (Without Dilated Convolution)	13.2 M	✓	×	90.33	96.36	95.81	77.54	78.99	65.31
			✓	78.59	98.39	96.64	78.57	80.27	67.08

R2U-Net: Recurrent Residual CNN based U-Net; MCGU-Net: Multi-level Context Gating; AA-UNet: Attention guided U-Net with Atrous convolution; DDNet: Dense Dilated Network; VGN: Vessel Graph Network; MTL: Multi-Task Learning; and SUD-GAN + : Short connection with Dense block-GAN.

Generally, the proposed CMM-Net provides effective segmentation results compared to ground-through manual annotations, as shown in Fig. 6. In addition, Fig. 6 presents the missing regions that are drawn with green contours. These missing regions represent the peripheral tiny vessels, which considered as a challenging task in the retinal vessels extraction. The results showed comparable performances between the proposed network and UNet +  + . In contrast, DeepLabv3 + could only extract the large blood vessels, while it failed to segment the small ones as shown in the same figure. This may be due to that DeepLabv3 + did not address the full use of the decoder, where the number of layers in the decoder (i.e., upsampling of 4 $\times$) are smaller than those in the encoder (i.e., downsampling of 0.5 $\times$), and that is why its number of trainable parameters are smaller compared to other methods.

#### Brain tumor segmentation performance

We also evaluated the proposed CMM-Net segmentation method using both the 57 local testing and the 66 actual validation MR subjects of the BraTS 2018 dataset. Table 5 summarizes the segmentation results of the proposed method compared to the top three ranked methods^78–84^ in the challenge besides the U-Net, PSPNet, DeepLabv3 + , CE-Net, and UNet +  + . The performance evaluation of this task was performed based on the volume segmentation level. Our proposed segmentation method outperformed the implemented U-Net and PSPNet based VGG network on the local testing data with a significant increment of 12.02% and 6.53% in term of the DSC, respectively. The proposed CMM-Net obtained slightly better DSC results compared to DeepLabv3 + , CE-Net, and UNet +  + with incremental rates of 0.67%, 1.05%, and 0.90%, respectively. Regarding the segmentation results of the actual validation data, we achieved overall segmentation sensitivity, specificity, and DSC scores of 96.21%, 99.77%, and 88.96%, respectively. It is of note that these results were obtained after submitting the segmented volumes of all validation data to the online submission system (https://ipp.cbica.upenn.edu/). The results of brain tumors segmentation via the proposed CMM-Net are efficient and comparable with the top three methods in the challenge with a slightly lower DSC of 88.96%. The challenge set the average of DSC indices for the WT, ET, and TC to rank the participants. However, here we focused only on the whole tumor segmentation as further evaluation of our proposed segmentation method. As reported in Table 5, the first ranked method in the challenge achieved a small increment rate of 2.04% in term of DSC compared to the proposed work. Comparable achievements were recorded for the proposed CMM-Net, DeepLabv3 + , CE-Net, and UNet +  + with DSCs of 88.96%, 88.04%, 88.57%, and 89.04% on the actual validation set, respectively. It is observed from Table 5 that the proposed network, CE-Net, and UNet +  + obtained lower segmentation performance when applying the IR process.

**Table 5:** Brain tumors segmentation performances (%) via the proposed CMM-Net compared to the recent deep learning approaches.

Test Set	Method	Parameters	Implemented	IR	SEN	SPE	ACC	MCC	DSC	JAC
Local Testing Set	U-Net^10^	7.3 M	✓	×	83.63	98.27	98.16	72.62	71.28	55.38
				✓	85.36	98.54	98.33	73.01	71.93	56.89
	PSPNet^15^	15.2 M	✓	×	74.94	**99.73**	**99.38**	77.29	76.89	63.91
				✓	87.52	99.43	99.27	77.88	77.42	63.92
	DeepLabv3 + ^17^	2.1 M	✓	×	81.30	99.30	99.03	83.45	82.86	72.42
				✓	89.43	99.10	98.96	84.02	83.28	73.40
	CE-Net^24^	26.0 M	✓	×	86.91	99.28	99.11	83.56	82.90	73.07
				✓	**90.57**	99.14	99.02	83.08	82.35	72.32
	UNet + + ^27^	24.2 M	✓	×	82.03	98.31	98.10	83.41	83.05	73.78
				✓	89.54	98.06	97.94	83.07	82.45	72.59
	Proposed CMM-Net	10.2 M	✓	×	85.19	99.55	99.35	**84.55**	**83.95**	**74.09**
				✓	90.48	99.28	99.17	82.63	82.01	72.23
Actual Validation Set	Encoder-Decoder based ResNet^78^ (1st place in the challenge)	-	×	×	-	-	-	-	91.00	-
	3D U-Net^79^ (2nd place in the challenge)	–	×	×	–	–	–	–	**91.26**	–
	Densely Connected DeepSCAN^80^ (3rd place in the challenge)	–	×	×	–	–	–	–	90.30	–
	Ensemble Network^81^ (3rd place in the challenge)	–	×	×	–	–	–	–	90.95	–
	HTTU-Net^82^	–	×	×	88.30	**99.90**	–	–	86.50	–
	AGResU-Net^83^	–	×	×	–	–	–	–	87.20	–
	3D U-Net^84^	–	×	×	88.70	99.50	–	–	88.90	–
	U-Net^10^	7.3 M	✓	×	80.02	99.79	–	–	73.18	–
				✓	84.77	99.71	–	–	74.50	–
	PSPNet^15^	15.2 M	✓	×	76.73	99.05	–	–	78.97	–
				✓	88.92	98.06	–	–	79.68	–
	DeepLabv3 + ^17^	2.1 M	✓	×	85.39	99.41	–	–	86.97	–
				✓	95.59	99.08	–	–	88.04	–
	CE–Net^24^	26.0 M	✓	×	90.03	99.27	–	–	88.57	–
				✓	92.69	98.93	–	–	87.68	–
	UNet + + ^27^	24.2 M	✓	×	87.59	99.50	–	–	88.96	–
				✓	93.03	99.05	–	–	89.04	–
	Proposed CMM-Net	10.2 M	✓	×	**96.21**	99.77	–	–	88.96	–
				✓	92.98	99.03	–	–	88.32	–

HTTU-Net: Hybrid Two Track U-Net; and AGResU-Net: Attention Gate Residual U-Net.

**Figure 7:**
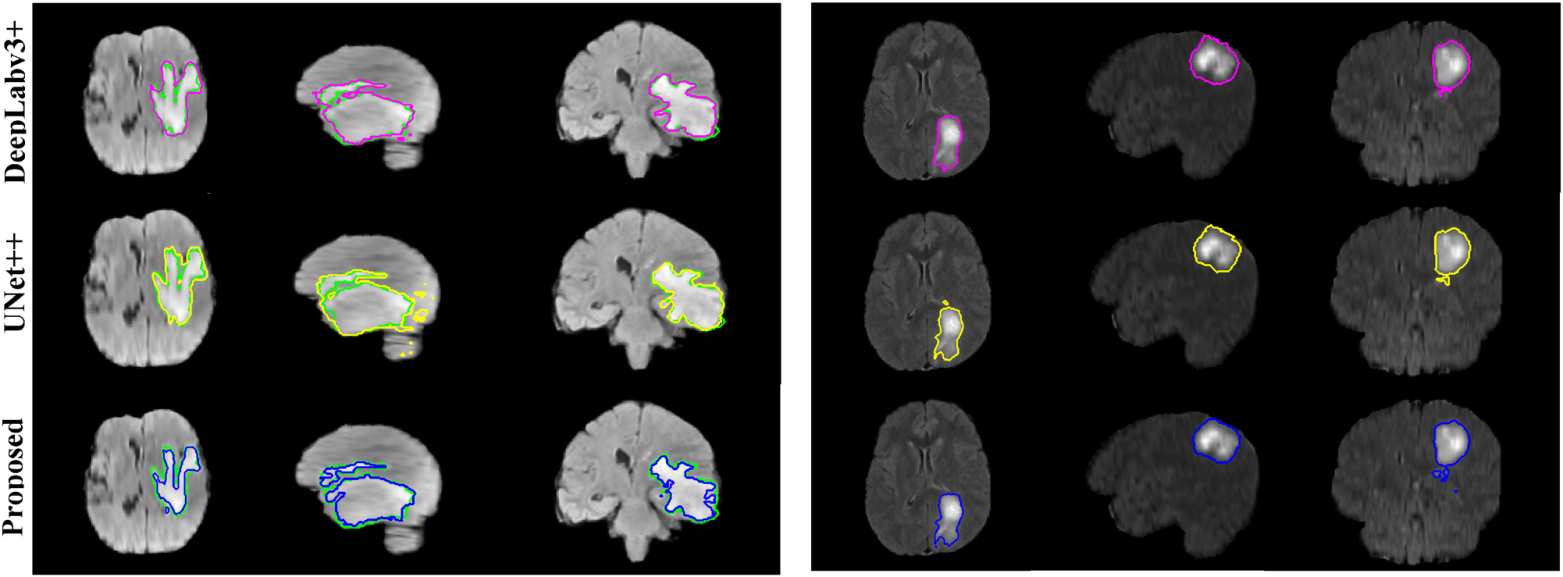
Examples of the segmented brain tumors. Left indicates some examples from the local test data, while right refers to examples from the actual validation data. The green, magenta, yellow, and blue contours refer to the ground-truth annotations and the segmented results via the DeepLabv3 + , UNet +  + , and proposed CMM-Net, respectively. First to third columns of each sample represent the axial, sagittal, and coronal views, respectively.

Figure 7 shows some segmentation examples of both local test data and actual validation data in three views (i.e., axial, sagittal, and coronal). Since the ground-truth annotations of validation data are not available, we only draw the segmented contours of the proposed method against DeepLabv3 + and UNet +  + . Obviously, the results indicate the capability of the proposed segmentation method in detecting the precise location of brain tumors. Our CMM-Net could efficiently differentiate between the gliomas and bright tissue regions that have high visual similarity.

### Overall evaluation

As a summary, the overall segmentation performances of skin lesions and retinal blood vessels were improved in the case of using the IR process. In the case of skin lesions, the proposed method achieved DSC of 83.13% without IR and 85.78% with IR. In the case of retinal blood vessels, the DSC also improved from 78.99% to 80.27% when applying the IR process. In contrast, this process did not provide improvement in the case of brain tumors segmentation neither for the local test where DSC dropped from 83.95% to 82.01% nor for the actual validation where DSC dropped from 88.96% to 88.32%. This may be due to that the structure of brain tumors is extremely irregular and in some cases exists as unconnected segments.

The obtained segmentation results demonstrate the efficiency of the proposed CMM-Net over different medical imaging tasks. Indeed, the aggregation of multi-scale contextual features throughout all networks’ encoder levels along with the dilated convolutions foster to learn more robust multi-spatial global representations with location-awareness. The ablation experiments shown in Table 2 presents the merit of using the PPMs as well as dilated convolutions in improving the performance. The generated multi-scale features at early levels of encoder fuse structural and localization details, while those at top levels enable to learn coarser information. It is of note that these multi-scale features fusion keep the computation low compared to other existing methods and at the same time provide rich contextual features. The overall segmentation performance of skin lesions was improved from 67.36% to 73.94% in term of Jaccard index when adopting the contextual multi-scale multi-level aggregation strategy. Compared to other existing methods in Table 3, the proposed CMM-Net achieved superior performance in the skin lesion segmentation task with an overall Jaccard index of 77.65%. Although comparable performances were obtained via the proposed CMM-Net and UNet +  + in the cases of retinal blood vessels and brain tumor segmentation tasks as reported in Tables 4 and 5, the amount of trainable parameters (i.e., computational cost) of the proposed method is around the half of that in UNet +  + . In addition to the reasonable computation cost compared to other existing works, generalization is one of the advantages of the proposed network. CMM-Net seems to be feasible solution for different medical imaging applications since it succeeds to segment three different medical tasks. In contrast, PSPNet and DeepLabv3 + failed to segment small or thin targets as the case of retinal blood vessels extraction.

**Figure 8:**
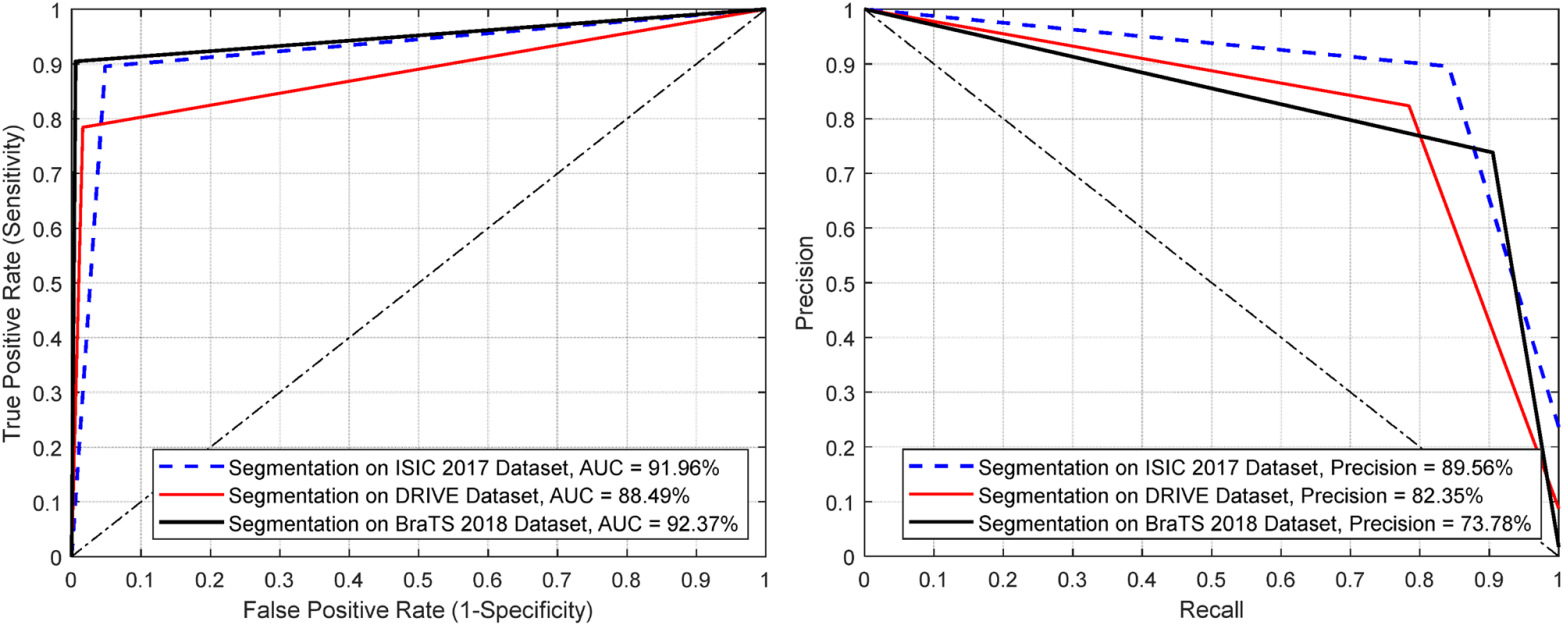
ROC curves (left) and PR curves (right) of our proposed CMM-Net segmentation method on three different medical applications.

**Figure 9:**
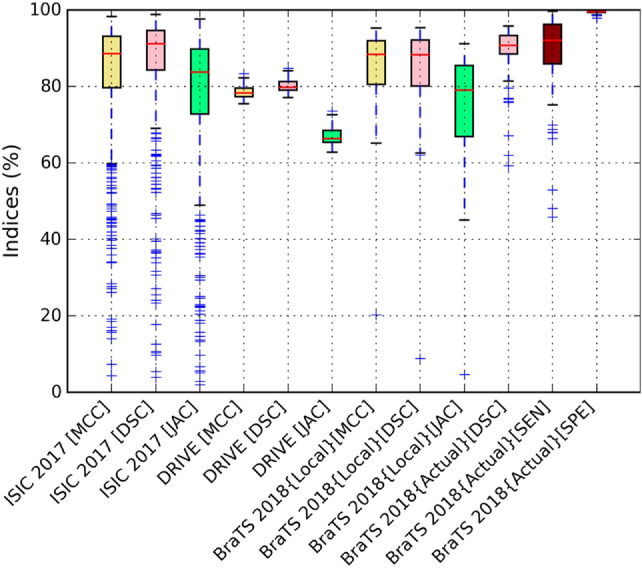
Boxplots of the segmentation performances of each testing data via the proposed CMM-Net.

Furthermore, Fig. 8 demonstrates the ROC and PR curves of the proposed CMM-Net for all the medical imaging segmentation applications. Our segmentation method obtained AUCs of 91.96%, 88.49%, and 92.37% for the skin lesions, retinal blood vessels, and brain tumors segmentation tasks, respectively. We illustrate the segmentation performance for each original input test data (i.e., images for the ISIC 2017 and DRIVE tasks, and patient volumes for the local test and actual validation in the BraTS 2018 case) in Fig. 9. This boxplot shows the MCC, DSC, and Jaccard indices for each test data. However, only the DSC, sensitivity, and specificity measures were obtained from the online evaluation for the actual validation data of BraTS 2018. Clearly, there is no high variation on the segmentation performances of the DRIVE and BraTS 2018 test datasets, while a small set from the test dermoscopy images of the ISIC 2017 dataset obtained less than 50% of the shown measures.

We also show the overall evaluations for all medical image modalities including skin lesions, retinal blood vessels, and local testing and actual validation sets of brain tumors in Table 6. The presented indices are computed as a weighted average based on the number of testing samples in each data. This evaluation shows the superior segmentation performance of the proposed CMM-Net with DSC of 85.77% against other networks. The proposed segmentation network presents its capability and efficiency to be generalized on segmenting different medical imaging applications. Overall, the proposed architecture is effective on several semantic segmentation tasks.

**Table 6:** Overall evaluation (%) for all medical image modalities.

Method	SEN	SPE	DSC
DeepLabv3 + ^17^	89.66	96.03	84.43
CE-Net^24^	83.62	97.80	84.03
UNet + + ^27^	88.76	96.33	84.93
Proposed CMM-Net	88.01	96.86	85.77

### Computation time

The training and inference (i.e., decoding or testing) computation times needed to accomplish the segmentation of different medical imaging applications via the proposed CMM-Net are listed in Table 7. As shown, the amount of the trainable parameters of the proposed network is higher in the case of the DRIVE dataset. This is due to that we were able to utilize larger feature maps of 128 for each PPMs in the case of retinal blood vessels dataset since the network was trained based on the smaller size of inputs (i.e., patches). However, the relevant required training time per each epoch is very large in the case of the BraTS 2018 dataset because it has the larger amount of the augmented training images of 122,320 images (i.e., 15,290 multiplied by eight augmented operations) with bigger input size compared to other datasets. As aforementioned in section III-A-2, the inference time for segmenting an entire retinal image required 0.45 s, which is computed as a multiplication of the testing time per each patch by the total number of patches. Then, the actual inference time of 3.6 s is computed by taking into consideration the number of augmented testing data that were used to apply the IR process. Similarly, the total required time to encode the whole brain volume is computed as a multiplication of 0.0253 s (i.e., the testing time per each slice image) by 155 (i.e., the total number of slices in each volume) without applying the IR. Further, the proposed method required a moderate amount of trainable parameters of 10.2 M compared to 15.2 M and 24.2 M in the cases of PSPNet and UNet +  + , respectively. Overall, the proposed CMM-Net seems to be feasible in the routine clinical exams.

**Table 7:** Training and testing computation times of our proposed CMM-Net for the three medical benchmarks.

Dataset	Image Size	Trainable Parameters	Training Time/Epoch (minutes)	Testing Time/Image (seconds)	Actual Testing Time/Input (seconds)	
					Without IR	With IR
ISIC 2017	192 $\times$ 256	10,246,562	43.72	0.033	0.033	0.792
DRIVE	128 $\times$ 128	13,221,794	4.75	0.018	0.45	3.6
BraTS 2018	192 $\times$ 192	10,246,562	86.4	0.0253	3.92	31.36

## Conclusion

This paper proposed a deep learning segmentation method called CMM-Net for three different medical imaging modalities. The proposed method exploited both the dilated convolution and pyramid pooling modules recurrently in the encoder network of the U-Net. Further, a modified evaluation scheme, called inversion recovery, that retrieves all the segmented images from the augmented testing data into a single output using logical operators is developed. The proposed segmentation method achieved superior performances on three different biomedical imaging tasks, including the segmentation of the skin lesions, retinal blood vessels, and brain tumors, compared to the recent deep learning approaches. The proposed method could be feasible for future medical imaging analysis and clinical exam routine. In the future, we plan to develop a 3D version of the CMM-Net along with the residual and dense mapping to be used for different types of brain tumors.

## Data Availability

Code of this work is available on GitHub through this link: https://github.com/Yonsei-MILab/Biomedical-Image-Segmentation-via-CMM-Net.
